# Quality improvement strategies for organizational change: a multiphase observational study to increase insight into nonparticipating organizations

**DOI:** 10.1186/s12913-018-3847-6

**Published:** 2018-12-29

**Authors:** Jeanny J. A. de Groot, Maite Timmermans, José M. C. Maessen, Bjorn Winkens, Carmen D. Dirksen, Brigitte F. M. Slangen, Trudy van der Weijden

**Affiliations:** 1 Department of Family Medicine, CAPHRI, School for Public Health and Primary Care, Maastricht University, P.O. Box 616, 6200 MD Maastricht, The Netherlands; 2 Department of Obstetrics and Gynaecology, Maastricht University Medical Centre, P.O. Box 5800, 6202 AZ Maastricht, The Netherlands; 3 Department of Research, Netherlands Comprehensive Cancer Organisation (IKNL), P.O. Box 19079, 3501 DB Utrecht, The Netherlands; 4 Department of Quality and Safety, Maastricht University Medical Centre, P.O. Box 5800, 6202 AZ Maastricht, The Netherlands; 5 Department of Methodology and Statistics, CAPHRI, School for Public Health and Primary Care, Maastricht University, P.O. Box 616, 6200 MD Maastricht, The Netherlands; 6 Department of Clinical Epidemiology and Medical Technology Assessment, CAPHRI, School for Public Health and Primary Care, Maastricht University Medical Centre, P.O. Box 5800, 6202 AZ Maastricht, The Netherlands; 7 GROW – School for Oncology and Developmental Biology, Maastricht University Medical Centre, P.O. Box 5800, 6202 AZ Maastricht, The Netherlands

**Keywords:** Implementation, Nonparticipant analysis, Awareness, Length of stay, Perioperative care

## Abstract

**Background:**

The scope of implementation research is often restricted to the analysis of organizations that participate voluntarily in implementation interventions. The recruitment of participants for a quality improvement collaborative increases awareness of the specific innovation. The objective of this multiphase observational study was to identify differences between organizations that participated in a large-scale implementation project aiming to improve perioperative care, functional recovery, and length of hospital stay after gynecologic surgery and organizations that did not participate. A secondary objective was to explore how perioperative practice changed among nonparticipants.

**Methods:**

Of the seven gynecology departments of nonparticipating Dutch hospitals, five agreed to participate in a retrospective analysis. Baseline data of participating hospitals’ (*N* = 19) characteristics, time to functional recovery, and length of hospital stay were compared. Outcome measures for the subsequent pre-post awareness study in the five nonparticipating hospitals were: (1) overall adherence to predefined evidence-based perioperative elements; and (2) change in functional recovery and length of hospital stay. Multivariable regression models, adjusted for baseline characteristics, were used for analysis.

**Results:**

In retrospect, nonparticipating and participating hospitals did not differ in baseline characteristics, functional recovery, and length of hospital stay. In three of the five nonparticipating hospitals, adherence to the selected evidence-based perioperative elements increased significantly after awareness of the trial (overall mean difference 9.7%, 95% CI 6.9 to 12.5%, *p* <  0.001). Linear regression models revealed no statistically significant or clinically relevant differences in time to functional recovery (mean difference − 0.2 days, 95% CI -0.7 to 0.2, *p* = 0.319) or length of hospital stay (mean difference − 0.4 days, 95% CI -1.3 to 0.5, *p* = 0.419) in the nonparticipating hospitals. None of these hospitals managed to reduce time to functional recovery or length of hospital stay significantly.

**Conclusions:**

No differences in perioperative outcomes between the nonparticipating and participating hospitals were identified at baseline. Despite the statistically significant improvement in overall evidence-based perioperative care, the awareness raised by recruitment activities alone was not enough to reduce time to functional recovery and length of hospital stay in nonparticipating hospitals. Insight into the trends of nonparticipants is valuable to existing implementation effectiveness research.

## Background

Several single and multifaceted implementation interventions have been used to improve the translation of knowledge and guidelines into medical practice [1–3]. These implementation interventions are specified activities designed to enhance adoption of clinical practice with the goal to improve healthcare outcomes [4, 5]. Their effectiveness in optimizing patient outcomes and healthcare quality has been addressed across different contexts, but further evaluations are still required in order to explore the practical implications of different strategies [2, 3, 6]. Whether beneficial implementation effects on health outcome or quality measures will occur and to what extent is difficult to predict in advance [2, 3, 6]. Moreover, the process of change requires the investment of considerable financial and personal resources [7, 8]. These considerations have the potential to influence health organizations’ choices regarding the execution of implementation interventions [2, 7].

Grol [9, 10] described several specific steps and related barriers involved in the process of change. Orientation, insight, and acceptance are necessary before actual change can begin in practice [10]. Studies have shown that organizational readiness facilitates the successful implementation of innovations and new guidelines [11]. In their review article, Weiner and colleagues conclude that both motivation and capability are essential components of readiness for change [11]. There has been increased awareness of the role of such contextual factors on the effectiveness of implementation interventions in healthcare [12, 13].

Achieving large-scale implementation of innovations and new guidelines across intra-organizational boundaries is even more complex. Quality improvement collaboratives are designed to improve healthcare quality and outcomes among multiple organizations by using a structured, temporary approach [6, 14]. Sponsorship and the type and priority of the specific issue might determine whether organizations participate in those collaboratives [15]. Participants usually take part in quality improvement collaboratives voluntarily in order to improve quality in healthcare [15]. This depends primarily on their willingness to invest and take part. The scope of implementation research is often restricted to the analysis of participants. Specific recruitment processes remain unexamined and could affect the representativeness of the study sample. Although previous studies have addressed nonparticipation in other settings, the evaluation of sample representativeness is particularly interesting in implementation research. In every process of change, target groups can be divided into several categories according to Rogers’s diffusion theory [16]. The process by which an innovation spreads among members of a social system over time is described in this conceptual theory. So, it can be assumed that participants of large-scale implementation strategies differ from nonparticipants. Analysis of demographic variables and reasons for nonparticipation could reveal subsequent participation bias.

In Grol’s ten-stage model [10], awareness is pointed out as the first orienting step in the process of change. This action model for effective implementation of change formulates a systematic step-wise proposal for change and can be used to plan and support an implementation process. The recruitment of participants in a quality improvement collaborative increases awareness of the specific innovation. We define awareness as a state of knowing and being informed about a clinical intervention to improve health care outcomes. It is assumed that awareness alone usually is not sufficient to change practice [17]. Evaluating the influence of this awareness in terms of how it effects change in medical practice among the nonparticipant group might further understandings of implementation processes. In the present study, we focused on the nonparticipants of the ‘Stepped Implementation of Enhanced Recovery in Gynecology’ (SINERGY) trial. The SINERGY trial is a nationwide comparative effectiveness implementation study that aims to compare two different large-scale implementation strategies across Dutch hospitals in order to define the most efficient strategy to improve practice [18]. A traditional breakthrough strategy with generic, short-term cyclic educational sessions is compared with an innovative stepped implementation strategy comprising different levels of intensity of support adapted to the specific needs of an organization. Reviews have indicated that implementing evidence-based perioperative elements in gynecologic surgery results in improved perioperative outcomes [19–21].

There were two primary objectives of the current study: (1) to identify differences between the nonparticipating and participating hospitals of the SINERGY trial; (2) to explore whether awareness of the start of a perioperative improvement program in other hospitals affected perioperative outcomes in hospitals that did not participate in the improvement program. The overall group effect on outcome measures and differences in effect within individual hospitals will be evaluated.

## Methods

### Design

A multiphase observational study was conducted sequentially. To assess the representativeness of the potentially eligible sample of the SINERGY trial, a retrospective observational multicenter approach was used, which compared nonparticipating hospitals with hospitals participating in the SINERGY trial. Subsequently, a pre-post study was conducted in the nonparticipating hospitals in order to identify any changes in perioperative practice, length of functional recovery, and length of postoperative hospital stay. This study was financially supported by the Netherlands Organization for Health Research and Development (ZonMw) (project number: 837003002).

### Setting

The current study can be seen as an addition to the nationwide SINERGY trial [18]. All the gynecology departments of the 27 hospitals authorized to perform major gynecologic oncologic surgery in the Netherlands were assessed for eligibility. In line with the SINERGY trial protocol [18], one hospital that referred most of its patients to inpatient rehabilitation facilities after hospital discharge was excluded a priori. In total, 26 eligible hospitals were invited to participate in the SINERGY trial.

### Recruitment process

A combination of targeted recruitment efforts were undertaken to increase involvement in the SINERGY trial. In mid-2013 local stakeholders were identified and contacted directly by members of the expert team. A personal letter was sent, which contained information about the study project, the implementation strategies being tested, the objectives of the perioperative guidelines, the need for active implementation, and the expected time and personal commitment required. In conformance with the SINERGY trial protocol [18], and in order to minimize implementation effects before the start of the study project, detailed information about the specific elements of the perioperative guidelines were not disseminated. Local stakeholders were, however, aware of the intentions of the SINERGY trial to reduce time to recovery and length of hospital stay by implementing evidence-based perioperative elements according to the enhanced recovery after surgery (ERAS) approach [18]. Reminders by email and telephone were used consecutively, when necessary. Visits to local organizations to provide additional information were undertaken on request. The study was supported by the Dutch Gynecology Oncology Group (DGOG), which comprises professionals who collaborate to promote research in gynecologic cancer. Funding provided by ZonMw helped to tackle financial disincentives by confining the local costs of implementation to attending meetings and executing local activities. Local stakeholders continued to be approached until a statement of commitment or nonparticipation was provided, resulting in a response rate of 100%. Even though all hospitals acknowledged the need for the nationwide standardization of perioperative practice consistent with ERAS, some decided not to participate in the SINERGY trial (nonparticipation rate 27%). These hospitals did not undergo a baseline audit of perioperative practice to determine if they were eligible for inclusion in the SINERGY trial. Stakeholders were asked about their reasons for nonparticipation. A combination of the compulsory nature of collective multidisciplinary meetings, a lack of time and resources due to high workload, and the lack of financial incentives were reported. In addition, nonparticipants questioned the return on their investment of implementing the evidence-based perioperative elements in improving time to recovery and length of hospital stay.

### Study groups

In total, seven hospitals chose not to take part in the SINERGY trial. Five of these seven hospitals agreed to participate in the current study. These ‘nonparticipating in SINERGY trial’ hospitals were compared with eligible participants of the SINERGY trial in order to address the first research objective of determining sample representativeness. The participant group consisted of hospitals that intended to participate in the SINERGY project (*N* = 19) [18]. For the second research objective, only the nonparticipating hospitals were analyzed for change in perioperative practice and outcomes following recruitment activities for the SINERGY trial. The comparison with participating hospitals will be made in future research, which will report data of the participating hospitals during the implementation interventions in more detail as well. Patients treated in the period before the recruitment process began, and thus before awareness of the SINERGY trial (pre-awareness group), were compared with patients treated one year after awareness of the trial (post-awareness group). This one-year period was chosen because the implementation interventions for the SINERGY trial were to begin in June 2014 [18]. In this way the effect of awareness was isolated as much as possible.

### Outcomes

Nonparticipating and participating hospitals were compared to identify differences in baseline characteristics before the onset of the recruitment process. The total number of beds per hospital, the type of hospital (university or non-university), and the level of specialized health care (secondary or tertiary) were obtained. Effect measures of the implementation of evidence-based perioperative elements were time to functional recovery and length of postoperative hospital stay. The outcomes in these effect measures were assessed in retrospect. Functional recovery was achieved when patients tolerated a normal diet, mobilized independently, and were comfortable on oral analgesia. Day zero refers to the day of surgery.

The outcomes of the second research objective, to explore the effect of awareness, were overall adherence rate to selected evidence-based perioperative care elements, time to functional recovery, and length of postoperative hospital stay in days. The selection of elements was in line with the SINERGY trial protocol [18] and an overall mean adherence rate was calculated. Selected elements included preadmission counseling and education, no bowel lavage, oral carbohydrate loading before surgery, routine use of prophylactic anti-emetics, nasogastric tube removal after surgery, no use of peritoneal drains, oral fluid intake on day zero, start solid food on the first postoperative day, mobilization on chair at least three times on the first postoperative day, use of epidural analgesia, routine use of oral laxatives, and urinary catheter removal before the third postoperative day. These predefined elements can be retrieved reliably and have a direct influence on recovery. In combination they should reflect an enhanced recovery approach.

### Data collection

In each nonparticipant hospital, retrospective data of two independent, consecutive samples of 30 patients were collected for the pre-awareness (January 2012) and post-awareness (June 2014) group. Baseline characteristics, surgical details, and outcome measures were retrieved from medical records. Patients underwent explorative laparotomies, surgical staging, or cytoreductive procedures for suspected, primary, or recurrent gynecologic malignancies. By selecting this specific group of patients, a comparable case-mix was provided for all hospitals. In addition, hospital characteristics were collected. For the participant group the baseline audit included a consecutive sample of 30 patients treated in 2012. No exclusion criteria were applied at the patient level.

### Data analysis

Absolute numbers with percentages were used for categorical data, while means with standard deviations (SD) or medians with ranges, where appropriate, were used for numerical variables. Differences were assessed using the independent-samples t-test or the Mann-Whitney U test for numerical variables, and the chi-square test or Fisher’s exact test, where appropriate, for categorical ones. Both unadjusted and adjusted statistical methods were used to identify differences in implementation endpoints between the nonparticipating and participating hospitals and to assess the overall effect of awareness on perioperative management and outcomes after elective gynecologic surgery. Linear multilevel analysis methods were used to compare the time to functional recovery and the length of hospital stay between the nonparticipating and participating hospitals. Models were adjusted for clustering (random effect) and patient and surgical characteristics (fixed effects). Study group, age, American Society of Anesthesiologists’ classification, (suspected) diagnosis and type of surgery, histological subtype, surgical approach, operation time, type of incision, and type of additional surgical procedure were included in the models as fixed effects. To evaluate the overall effect of awareness, the hospitals included in the nonparticipant study could not be treated as a random sample, but were included in the model as a fixed factor. So, multivariable linear regression models, adjusted for patient and surgical characteristics, were used. Furthermore, we were also interested in the separate outcomes for each hospital. Therefore, subsequent analyses of interaction terms between groups (pre-awareness versus post-awareness) and hospitals were performed to determine separate effects. Adjusted mean differences (MD) and 95% confidence intervals (CI) for results in linear regression models were reported. Multiple imputations (20 imputed datasets) were used to replace missing data on covariates, where appropriate. Since there was only one case with a missing covariate in both the pre-awareness and post-awareness group, complete case analysis was used for the regression models of nonparticipants. In a sensitivity analysis, logarithmic transformation of the data on time to functional recovery and length of postoperative hospital stay was used to analyze the influence of right-skewness of data in the nonparticipant analysis. A *p*-value ≤0.05 was considered statistically significant. A more than one day reduction in time to functional recovery and length of hospital stay (pre-awareness versus post-awareness) were considered clinically relevant. Analyses were performed using IBM SPSS Statistics for Windows, version 21.0 (Armonk, NY, USA).

## Results

### Nonparticipating versus participating hospitals

A total of 24 hospitals were compared, five nonparticipating and 19 participating. Although the nonparticipating hospitals tended to be smaller, no significant differences in the baseline characteristics of the nonparticipating and participating hospitals were found (Table 1). Unadjusted analysis demonstrated no significant differences between hospital groups in the SINERGY trial effect outcomes, that is, in time to functional recovery (*p* = 0.205) and length of postoperative hospital stay (*p* = 0.659) at baseline (Table 1). Linear multilevel analysis of time to functional recovery demonstrated an adjusted mean difference of 0.3 (95% CI -0.2 to 0.8) days (*p* = 0.256) between the nonparticipating and the participating hospitals. The adjusted mean difference in length of postoperative hospital stay was 0.0 (95% CI -0.8 to 0.8) days (*p* = 0.977).

**Table 1:** Baseline characteristics of participating and nonparticipating hospitals to the SINERGY trial (*N* = 24)

		Participating hospitals (*N* = 19)	Nonparticipating hospitals (*N* = 5)	*p* value
Total number of hospital beds, median [range]		715 [367–1320]	653 [180–976]	0.455^a^
Type of hospital, N (%)				> 0.999^b^
	University medical center	6 (31.6)	1 (20.0)	
	Non university teaching hospital	13 (68.4)	4 (80.0)	
Level of care, N (%)				0.631^b^
	Secondary care	12 (63.2)	4 (80.0)	
	Tertiary care	7 (36.8)	1 (20.0)	
Time to functional recovery (days)				
	Unadjusted, median [range]	4.0 [3.0–5.0]	5.0 [3.0–6.0]	0.205^a^
Length of postoperative hospital stay (days)				
	Unadjusted, median [range]	6.0 [4.0–10.5]	5.0 [3.0–7.0]	0.659^a^

Group differences were tested using the Mann-Whitney U test ^a^ or Fisher’s exact test ^b^ at hospital level. N = number of hospitals

### Pre-awareness versus post-awareness group

In the nonparticipating hospitals, the medical records of a total of 300 patients who underwent elective gynecologic surgery were collected for the pre-awareness (*n* = 150) and post-awareness (n = 150) groups. Patient characteristics and surgical details for the patients treated at baseline (pre-awareness) and post-awareness are shown in Table 2. Characteristics were similar in terms of American Society of Anesthesiologists’ classification, (suspected) diagnosis, and histological subtype; however, mean (± SD) patient age was significantly higher in the post-awareness group (61.8 ± 12.2 versus 65.1 ± 12.7 years, *p* = 0.024). Surgical approach, length of surgery, and the presence of additional procedures did not differ statistically between the study groups. More patients in the post-awareness group underwent primary cytoreductive surgery for ovarian cancer rather than an explorative or staging procedure compared to the pre-awareness group (*p* = 0.027).

**Table 2:** Baseline characteristics and surgical details of the pre-awareness and post-awareness groups

		Pre-awareness (*n* = 150)	Post-awareness (*n* = 150)	*p* value
Age in years, mean ± SD		61.8 ± 12.2	65.1 ± 12.7	0.024^a^
ASA classification, n (%)				0.757^b^
	I/II	124 (82.7)	126 (84.0)	
	III/IV	26 (17.3)	24 (16.0)	
(Suspected) diagnosis, n (%)				0.110^b^
	Ovarian cancer	115 (76.7)	114 (76.0)	
	Uterine cancer	27 (18.0)	34 (22.7)	
	Cervical cancer	8 (5.3)	2 (1.3)	
Histological subtype, n (%)				0.111^b^
	Benign	37 (24.7)	27 (18.0)	
	Borderline / hyperplasia	14 (9.3)	8 (5.3)	
	Malignant	99 (66.0)	115 (76.7)	
Surgical approach, n (%)				> 0.999^c^
	Midline incision	147 (98.0)	147 (98.0)	
	Transverse incision	3 (2.0)	3 (2.0)	
Operation time in minutes, mean ± SD		138.9 ± 72.0	142.7 ± 67.5	0.632^a^
Type of surgery *(only ovarian included)*, n (%)				0.027^b^
	Explorative	50 (43.5)	40 (35.1)	
	Staging	13 (11.3)	6 (5.3)	
	Primary cytoreductive	11 (9.6)	25 (21.9)	
	Secondary cytoreductive	41 (35.7)	43 (37.7)	
Additional surgical procedure, n (%)				
	Open-close (inoperable)	1 (0.7)	5 (3.3)	0.214^c^
	Anastomosis	6 (4.0)	7 (4.7)	0.777^b^
	Diverting stoma	6 (4.0)	9 (6.0)	0.427^b^
	Lymphadenectomy	34 (22.7)	27 (18.0)	0.315^b^
	Extensive upper abdominal surgery *	4 (2.7)	8 (5.3)	0.239^b^

Group differences were tested using the independent samples t-test ^a^, Chi-square test ^b^, or Fisher’s exact test ^c^. ASA = American Society of Anesthesiologists, n = number of patients, SD = standard deviation. * Includes diaphragmatic stripping, partial liver resection, or splenectomy

**Fig. 1:**
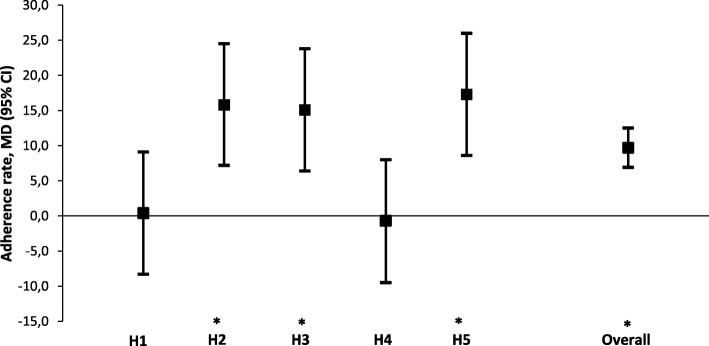
Title: Mean differences in adherence rate to the preselected perioperative elements per hospital (pre-awareness versus post-awareness) Legend: Multivariable linear regression models were adjusted for age, ASA classification, diagnosis and type of surgery, histological subtype, surgical approach, duration of surgery, and type of additional surgical procedure. Hospital identifiers were entered in the models as dummy variables with interaction terms. * indicates statistically significant difference. CI = confidence interval, MD = mean difference

Adherence rates to the perioperative elements of the ERAS pathway pre- and post-awareness are shown in Table 3. Anti-emetics were routinely used during surgery in 61.6% (*n* = 90) of patients in the pre-awareness group compared with 74.7% (*n* = 112) of patients in the post-awareness group (*p* = 0.016). In addition, a significant increase was observed in adherence rates for the avoidance of intraperitoneal drains (69.3% versus 81.9%, *p* = 0.011), early oral intake of fluids (24.7% versus 42.6%, *p* = 0.001) and solid food (55.3% versus 77.7%, *p* <  0.001), and early mobilization on postoperative day one (14.1 versus 27.3%, *p* = 0.005). On the other hand, slightly more patients were pretreated with oral bowel lavage solutions (0.0% versus 5.4%, *p* = 0.003). Overall, there was a significant difference in unadjusted adherence rates to the 12 predefined perioperative elements between the pre- and post-awareness group (mean rate 46.1 ± 15.5 versus 53.3 ± 13.3, *p* <  0.001) (Table 4). Multivariable linear regression, adjusted for baseline characteristics and surgical approach, demonstrated a similar result (MD 9.7, 95% CI 6.9 to 12.5%, *p* <  0.001). A significant increase in the adjusted mean difference in adherence rate was observed for three out of five hospitals (Hospital 2: *p* <  0.001, Hospital 3: *p* = 0.001, and Hospital 5: *p* <  0.001) (Fig. 1). There was no significant difference in adherence rates to the predefined perioperative elements between the two study groups for Hospital 1 (*p* = 0.921) and Hospital 4 (*p* = 0.875) (Fig. 1).

**Table 3:** Adherence rates to the selected perioperative care elements

			Pre-awareness (n = 150)	Post-awareness (n = 150)	*p* value^a^
Preoperative items, n (%)					
	Counseling and education		0 (0)	0 (0)	
	No bowel lavage		150 (100)	140 (94.6)	0.003^b^
	Oral carbohydrate loading (if indicated)		32 (21.3)	35 (23.3)	0.782
Intraoperative items, n (%)					
	Epidural analgesia		121 (80.7)	126 (84.0)	0.451
	Routine anti-emetics		90 (61.6)	112 (74.7)	0.016
	No use of drains		104 (69.3)	122 (81.9)	0.011
	Nasogastric tube removal		105 (70.0)	112 (75.7)	0.272
Postoperative items, n (%)					
	Avoidance of ileus				
		Oral laxatives at day 1	33 (22.0)	42 (28.0)	0.232
	Early oral intake				
		Oral fluids intake day 0	37 (24.7)	63 (42.6)	0.001
		Solid food intake ≤ day 1	83 (55.3)	115 (77.7)	< 0.001
	Early mobilization				
		On chair (minimum 3 times) ≤ day 1	21 (14.1)	41 (27.3)	0.005
	Early removal of catheters				
		CAD removal ≤ day 2	51 (34.2)	47 (31.5)	0.623

Values are valid percentages (excluding missing values). ^a^ Chi-square test. ^b^ Fisher’s exact test. CAD = urinary catheter, n = number of patients

**Table 4:** Unadjusted and adjusted outcomes for the pre-awareness and post-awareness groups

		Pre-awareness (*n* = 150)	Post-awareness (*n* = 150)	Mean difference (95% CI)	*p* value
Adherence to key items (%)					
	Unadjusted mean ± SD	46.1 ± 15.5	53.3 ± 13.3	7.3 (4.0 to 10.5)	< 0.001^a^
	Adjusted EMM (95% CI)	54.3 (41.5 to 67.1)	64.0 (51.2 to 76.9)	9.7 (6.9 to 12.5)	< 0.001^b^
Time to recovery (days)					
	Unadjusted mean ± SD	4.8 ± 2.4	4.9 ± 2.2	0.0 (−0.5 to 0.6)	0.892^a^
	Adjusted EMM (95% CI)	4.9 (2.7 to 7.0)	4.6 (2.5 to 6.8)	-0.2 (−0.7 to 0.2)	0.319^b^
Length of hospital stay (days)					
	Unadjusted mean ± SD	6.4 ± 4.2	6.5 ± 4.1	0.1 (−0.9 to 1.0)	0.874^a^
	Adjusted EMM (95% CI)	6.6 (2.6 to 10.7)	6.3 (2.2 to 10.3)	−0.4 (−1.3 to 0.5)	0.419^b^

Group differences were tested using the independent samples t-test ^a^, or multivariable linear regression models ^b^. Linear models were adjusted for age, ASA classification, diagnosis and type of surgery, histological subtype, surgical approach, duration of surgery, and type of additional surgical procedure. Analysis of the log-transformed data on time to recovery and length of hospital stay showed similar results. CI = confidence interval, EMM = estimated marginal mean, n = number of patients, SD = standard deviation

**Fig. 2:**
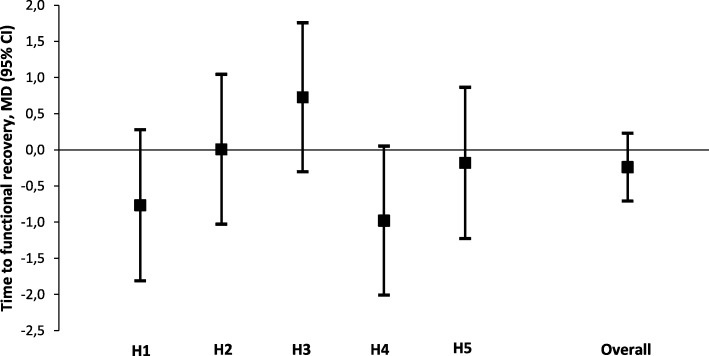
Title: Mean differences in time to functional recovery (days) per hospital (pre-awareness versus post-awareness) Legend: Multivariable linear regression models were adjusted for age, ASA classification, diagnosis and type of surgery, histological subtype, surgical approach, duration of surgery, and type of additional surgical procedure. Hospital identifiers were entered in the models as dummy variables with interaction terms. Analysis of the log-transformed data showed similar results. No statistically significant differences were found. CI = confidence interval, MD = mean difference

Functional recovery data were obtained for 294 patients (98.0%). In the pre-awareness group three patients died during admission (2.0%). In the post-awareness group one patient died shortly after discharge (0.7%) (*p* = 0.622). The mean (± SD) time to functional recovery was 4.8 ± 2.4 days in the pre-awareness and 4.9 ± 2.2 days in the post-awareness group (*p* = 0.892) (Table 4). Adjusted analysis of time to functional recovery demonstrated a non-significant reduction of 0.2 days (95% CI -0.7 to 0.2, *p* = 0.319) (Table 4). The effect of awareness on time to functional recovery did not differ significantly for any hospital (Fig. 2).

**Fig. 3:**
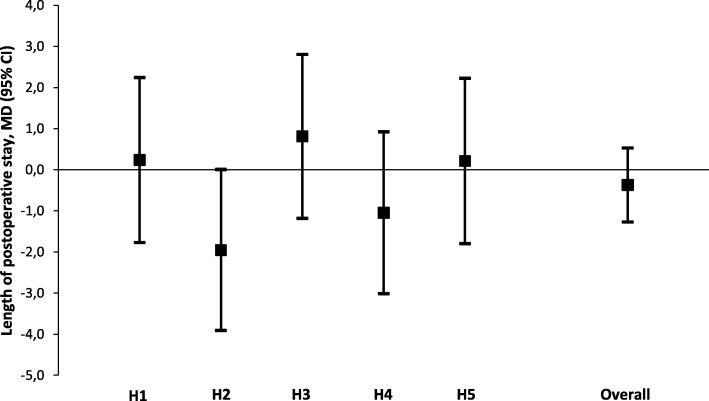
Title: Mean differences in length of postoperative stay (days) per hospital (pre-awareness versus post-awareness) Legend: Multivariable linear regression models were adjusted for age, ASA classification, diagnosis and type of surgery, histological subtype, surgical approach, duration of surgery and type of additional surgical procedure. Hospital identifiers were entered in the models as dummy variables with interaction terms. Analysis of the log-transformed data showed similar results. No statistically significant differences were found. CI = confidence interval, MD = mean difference

No statistically significant or clinically relevant difference in the unadjusted mean length of hospital stay after surgery was observed between the pre- and post-awareness group (6.4 ± 4.2 versus 6.5 ± 4.1 days, *p* = 0.874) (Table 3). Multivariable linear regression showed that none of the hospitals had a statistically significant change in the length of postoperative hospital stay (Fig. 3). The overall adjusted mean difference remained non-significant (MD − 0.4 days, 95% CI -1.3 to 0.5, *p* = 0.419) (Table 4).

## Discussion

In this study, we had the opportunity to illustrate the characteristics of and changes in organizations that did not participate in a nationwide comparative effectiveness implementation trial to implement evidence-based perioperative guidelines across gynecology departments in the Netherlands [18]. The findings provide insight into the effect that awareness of the start of an implementation program can have on the perioperative outcomes of hospitals not participating in the improvement program.

The current study found a significant change in overall perioperative practice, resulting in an estimated mean adherence of 64.0% (95% CI 51.2 to 76.9%) to the selected evidence-based elements in the post-awareness group. Analysis of the individual perioperative elements showed that more routine anti-emetics were administered and less intraperitoneal drains were used during surgery. In addition, an increase in early oral intake and early mobilization rates was found. Nevertheless, no statistically significant or clinically meaningful reduction in time to functional recovery or length of postoperative hospital stay occurred in the post-awareness group (MD − 0.2 days, *p* = 0.319 and MD − 0.4 days, *p* = 0.419, respectively). These results support previous research findings regarding guidelines for enhanced recovery after surgery, which indicate a 70% cutoff as the minimum level of adequate adherence [22, 23]. The enhanced recovery after surgery guidelines developed by the ERAS Society® include over 20 elements that, according to the best evidence available, are necessary for optimal perioperative care [24, 25]. The guidelines are used to reduce the gap between research and practice and aim to minimize the stress response to surgery [26]. As a result, functional recovery and time to discharge are supposed to improve. In accordance with the SINERGY trial protocol [18], we analyzed adherence to 12 of these perioperative elements. These predefined elements can be retrieved reliably and have a direct influence on recovery. In combination they should reflect an enhanced recovery approach. The elements were equally weighted to calculate the adherence rate as a measure of the implementation process. Perioperative data were obtained retrospectively through the review of medical records, which may have introduced bias to the results.

On a hospital level, three out of five hospitals achieved a significant improvement in evidence-based perioperative care. However, none of them reached the 70% cutoff and none managed to reduce the effect outcomes on time to functional recovery and length of hospital stay significantly. So, while there is some evidence that local stakeholder awareness of this intended change in perioperative practice might have affected the implementation of single evidence-based elements in nonparticipating hospitals, the improvement did not significantly or clinically change time to functional recovery or length of postoperative hospital stay. These findings are in agreement with Grol’s theory regarding the several steps involved in the process of change [10]. Merely increasing awareness through external peers during recruitment activities does not seems sufficient in starting the cascade of change needed to implement complex, multimodal innovations.

Based on Rogers’s diffusion theory [16], participating organizations probably belong to the early adopter and early majority categories. These organizations already have the best starting position for achieving beneficial implementation effects compared to nonparticipating organizations. The lack of clinical effect might be explained by differences in resources and willingness to change. Research on the effect of strategies used to implement innovations or guidelines should, therefore, not present data about the volunteering study sample only [27]. Additional information about nonparticipating organizations and contextual factors is particularly necessary to provide representative effect outcomes of different implementation strategies [27]. Mays et al. [28] showed that the public health organizations involved in research networks tend to be more engaged in implementation activities. Based on their findings, the authors suggest that the success of research networks is not only due to selection bias during the recruitment phase, but also to the development of competences during participation in research networks [28]. The findings of this quantitative study do not give insight into the underlying processes involved. Therefore, the limited effect could be due to the combination of a preexisting resistance to change and to a lack of resources and tools for achieving organizational change.

Organizational readiness seems to be essential in the process of change [11]. On the other hand, components of organizational readiness determine whether organizations become participants in implementation interventions [15]. In the beginning, we intended to include all eligible hospitals in the SINERGY trial. The process of recruiting hospitals was slow and difficult, despite the use of targeted strategies and facilitators. An evidence-based innovation that had already been proved effective, local stakeholders, peer pressure, and general funding of implementation strategies were used to increase participation. Eventually, a participation rate of 73% of all eligible hospitals was achieved. Stakeholders stated that their decision not to participate was based on organizational and financial constraints. This is in line with factors identified by a recent qualitative report [29]. In retrospect, because baseline outcomes in effect measures did not differ between participating and nonparticipating organizations they appear not to have influenced the choice to participate in implementation interventions. Prior studies have determined the importance of addressing local barriers to change during implementation interventions [9]. To increase organizational readiness and the extent of implementation effects, barriers for participating in large-scale implementation interventions also have to be taken into account. In addition, targeted implementation interventions based on specific organizational needs might be effective in increasing participation rates. Organizational readiness is difficult to quantify, therefore qualitative data could have provided more information. Besides, this could have helped to interpret quantitative findings.

Our findings give insight into the characteristics of and changes that took place in organizations that did not participate in a nationwide comparative effectiveness trial, but they must be interpreted with some caution. Despite the nationwide focus, only a relatively small number of hospitals could be included because of the specific study design. Multivariable regression analyses, with the hospital as a fixed instead of random factor, were used to explore the influence of awareness on perioperative care and outcomes. Therefore, the results are specific to our study sample and regression outcomes lack statistical generalizability. Regardless, we think that the perceived challenges of the recruitment phase and the observed trends in our study might be illustrative of other situations as well, particularly due to its nationwide focus. Data were retrieved from a consecutive sample of patients per hospital. The sample size of 30 patients per hospital was mainly based on the volume norm for Dutch hospitals. To increase comparability between hospitals all patients included in the study underwent surgery within the same one-year period. Inclusion of a larger number of patients per hospital would have resulted in a longer study period, thereby increasing the chance of bias.

## Conclusions

This multiphase observational study has shown that neither hospital characteristics nor perioperative outcomes at baseline differed, in retrospect, between the participating and nonparticipating departments of a national large-scale implementation project. Although a statistically significant improvement in evidence-based perioperative practice was achieved over time, the awareness raised by recruitment activities alone was not enough to facilitate change in functional recovery and length of hospital stay in the nonparticipating hospitals. Despite its exploratory nature, this study offers some insight into the influence of large-scale implementation projects on clinical practice in nonparticipating hospitals.

## Abbreviations

CI: confidence intervals
DGOG: Dutch Gynecology Oncology Group
ERAS: enhanced recovery after surgery
MD: mean differences
SD: standard deviation
SINERGY: Stepped Implementation of Enhanced Recovery in Gynecology
ZonMw: the Netherlands Organization for Health Research and Development.
